# Challenges and Neuropsychological Functioning in Children and Adolescents with Borderline Intellectual Functioning

**DOI:** 10.3390/children9121847

**Published:** 2022-11-28

**Authors:** Heli Sätilä, Laura Mirjami Jolma, Mira Meriläinen-Nipuli, Mikko Koivu-Jolma

**Affiliations:** 1 Department of Child Neurology, Päijät-Häme Central Hospital, Keskussairaalankatu 7, 15850 Lahti, Finland; 2 Faculty of Medicine, University of Helsinki, Haartmaninkatu 8, 00014 Helsinki, Finland; 3 Department of Child and Adolescent Rehabilitation, Päijät-Häme Welfare Area, Aleksanterinkatu 11, 15110 Lahti, Finland; 4 Faculty of Science, University of Helsinki, Gustaf Hällströminkatu 2, 00014 Helsinki, Finland

**Keywords:** cognitive development, borderline intellectual functioning, intellectual disability, neurodevelopmental disorders, learning disabilities, neuropsychological functioning, children, adolescents, young adults, psychiatric comorbidities

## Abstract

This retrospective chart review study sought to explore neuropsychological profiles, neuropsychiatric and psychiatric comorbidity, changes in diagnoses, support at daycare and school, medication use, psychiatric referrals, and progression into further education in a cohort of participants with borderline intellectual functioning (BIF). Additionally, developmental factors connected to BIF were studied. Delays in language and gross motor development were the initial reasons for the parents to seek health care. Comorbid neuropsychiatric and psychiatric diagnoses were frequent, a total of 41% of participants were referred to psychiatric services, and 45% used medication. Educational support was needed by 92% of the study participants. The majority of those graduating elementary school continued their studies at ordinary or special vocational schools. The risk of dropping out during secondary studies appeared to increase. The results in most of the neuropsychological subdomains declined over time, and 23% of the participants were later diagnosed with an intellectual disability (ID). The early developmental signs pointing towards BIF and the need for prompt support were a delay in language and motor development, difficulties in executive function, a delay in learning the activities of daily living among children under school age, and difficulties in reading and arithmetic skills and abstract reasoning at school age. It is important to follow up and support individuals with BIF as their risk for being left behind in the society is increased. Also, it would be important to repeat the neuropsychological testing of cognitive and adaptive functions before graduating elementary school as to capture those who meet the ID criteria.

## 1. Introduction

Borderline intellectual functioning (BIF) is defined as a neurodevelopmental disorder situated between normal cognitive functioning and mild intellectual disability (ID), corresponding to an intelligence quotient (IQ) test score of one to two standard deviations below average in the range of 70 to 85 [1,2]. BIF has also been called “slow learning”, “general learning disability”, “global developmental delay or disorder”, or “mild cognitive impairment”, referring to individuals with difficulties in adaptive behavior that affect every area of life but that do not fulfil the diagnostic criteria for ID [1,2]. More precisely, the diagnosis of “global developmental delay” is reserved for children under the age of 5, some of whom may later meet the diagnostic criteria for ID [3]. Thus, BIF may serve as an interim diagnosis before another more specific diagnosis or ID is clarified. In the International Classification of Diseases (ICD-10) [4], BIF does not have a clear definition but the codes F83 (mixed specific developmental disorders) and F81.3 (mixed disorder of scholastic skills) are used with children and those of school age with deficiencies in more than two neurodevelopmental (gross and fine sensorimotor, language, cognition, socioemotional, or activities of daily living) or learning (verbal, visual/perceptional, reading, calculating, memory, attention, or executive functioning) domains. In the Diagnostic and Statistical Manual of Mental Disorders (DSM-5) [5], diagnosis does not lean on IQ scores only, but emphasizes the differentiation of ID and BIF by assessing the discrepancies of cognitive and adaptive function.

Depending on criteria, the overall prevalence of BIF is estimated to be 7–14% [2,6,7]. The risk factors and etiology vary from perinatal causes and genetic factors to maternal and environmental factors such as fetal alcohol spectrum disorder, child neglect, and deprivation [8,9,10,11,12,13,14,15,16]. Comorbidities such as attention-deficit hyperactivity disorder (ADHD), depression, anxiety, autistic features or autism spectrum disorder, or difficulties in conduct and social interaction, are frequent and may complicate performance in school, employment, and life in general [17,18,19,20,21,22,23]. About 40% of children and adolescents with BIF have been reported to have a psychiatric comorbid disorder, and about 30% of these diagnosed patients received mental health care [24]. Adults with BIF are at increased risk of mental health disorders, substance use, unemployment, and antisocial or criminal behavior [25,26,27,28,29,30].

Neuropsychological profiles and difficulties in cognitive skills and adaptive behaviors are heterogeneous. Individuals with BIF have been reported to have deficiencies in their academic skills (such as arithmetic, reading comprehension, and mechanical reading), cognitive skills (such as working memory in both the verbal and visuo-spatial domains, processing speed, learning strategies, abstract mentalization, and executive function), social skills (such as recognizing and verbalizing emotions, theory of mind skills, social interaction and participation, social information processing, and recognizing facial expressions), practical adaptive skills (activities of daily living), and motor skills [1,2,11,31,32,33]. The severity of difficulties may vary between developmental domains from mild to severe, and the profiles may be versatile even in the same individual. Usually, mechanical skills are well managed, but abstract thinking, executive functioning, and adaptive problem solving are weak [2,32]. However, some individuals with this disorder may have good daily living skills.

Despite the prevalence of BIF in pediatric psychiatric and neurological settings, it is a rarely studied topic, and this population has remained a marginal clinical entity with vague diagnostic and therapeutic approaches [1,32]. The purpose of this retrospective chart review study was, therefore, to explore the neuropsychological profiles, neuropsychiatric and psychiatric comorbidities, changes in diagnoses, use of therapies, support needed at daycare and school, medication use, need for psychiatric referrals, and progression into further education and employment in a cohort of children and adolescents with BIF. Additionally, developmental factors connected to BIF were studied.

## 2. Materials and Methods

*Subjects.* This retrospective chart review study was conducted on the patient registers of 651 children and adolescents who visited the multidisciplinary neuropediatric developmental clinic between January 2010 and December 2020 and received an ICD-10 diagnosis of F83 or F81.3 at some point during the assessments. Inclusion criteria were an age over 5 years at the timepoint of data extraction and documented clinical history of physical, language, occupational, and neuropsychological evaluations. The study was conducted according to the guidelines of the Declaration of Helsinki and approved by the local research ethic committees (Helsinki University Hospital/Project identification code: HUS 3134_2020; and Päijät-Häme Central Hospital/Project identification code: D/2603/07.01.04.05/2019).

*Past history.* The data extracted from the patient records comprised the following: birth, maternal pregnancy, and family history; the age when first consulted because of developmental delays; the types of therapies and support received; the psychiatric, neurological, and neuropsychiatric co-morbid diagnoses; the changes in neuropsychological cognitive profiles and adaptive skills; the daily living skills; the type of support needed at the daycare or school (e.g., special education); the need for psychiatric or social child welfare care referrals; the use of neurological or psychiatric medication; and academic educational or vocational success (or non-success). A possible change in diagnosis during the developmental course was noted. All of data were not available for every patient in the analysis of neuropsychological and neurodevelopmental subgroup profile changes, thus the size of the groups varied.

*Assessments.* Patients under school age were evaluated by a neuropediatric multidisciplinary group (pediatric neurologist, neuropsychologist, speech therapist, occupational therapist, and physiotherapist). The diagnosis of F83 was given when there was a delay of 1.5 or more standard deviations below the mean of norm-referenced standardized tests in two or more developmental domains (gross and fine sensorimotor, language, cognition, socioemotional, or activities of daily living). The school-aged individuals with learning difficulties were evaluated by a neuropsychologist (either by the preschool/school psychologist or by the multidisciplinary group neuropsychologist) and a pediatric neurologist. A diagnosis of F81.3 was given when two or more cognitive domains were delayed by at least 1 to 2 standard deviations below the mean of norm-referenced standardized tests but did not fulfill the ID criteria, and the child’s situation was not caused by a poor learning environment or psychiatric disorder. The standardized tests used by the neuropsychologists were as follows: the Neuropsychological Test for Children Finnish version (NEPSY-II); Wechsler Preschool and Primary Scale of Intelligence (WPPSI-III)/Wechsler Intelligence Scale for Children (WISC-IV); and, when appropriate, ADHD Rating Scale-IV, Social Responsiveness Scale Finnish version (SRS), and Strengths and Difficulties Questionnaire (SDQ-Fin). Comorbidities such as ADHD or autism spectrum disorder were diagnosed according to the ICD-10 and Finnish Best Practice Recommendations. The tests for each participant were chosen individually by the assessing neuropsychologist according to the age and profile of the child. An important part of the diagnosing process and rehabilitation follow-up was the written information given by the parents, day care personnel or schoolteachers, and the child’s personal therapists.

*Statistical analysis*. R 4.1.3 (R Core team 2022) was used for statistical analysis. Correlations were calculated using the R package Hmisc (Harrell 2022). All of data were not available for every patient in all categories, thus the size of the groups varied from test to test. The descriptive statistics were given for the whole group. In analysis, the NEPSY-II neuropsychological test subdomains attention, executive functions, impulsiveness, reading skills, arithmetic skills, abstract concepting skills, conduct, self-esteem, and general performance in life skills, were categorized as “major difficulties”, “minor difficulties”, “no difficulties”, or “not evaluated”. The proportions of patients with “major difficulties” test results in the subdomains at the first and last assessment session were compared using 2-sample test for equality of proportions with continuity correction. Χ^2^ test statistic and p-values were used as measures of statistical significance. The WPPSI-III/WISC-IV “total cognition” and it´s subdomains “verbal performance”, “visual performance”, “processing speed”, and “working memory” were categorized as “Extremely Low” (below 70), “Borderline” (70–79), “Low Average” (80–89), “Average” (90–109), “High Average” (110–119), “Superior” (120–129), or “Very Superior” (130–), because the IQ scores from most of the previous neuropsychological tests performed outside our institution were not available. The change in frequencies was analyzed. Because the test results are non-continuous and non-linear, non-parametric Spearman’s ρ was chosen to measure the correlations of the elapsed time between the two assessment sessions and the changes in the results. The gross motor function, fine motor function, balance, eye-hand coordination, sensory processing, play skills, social interaction, and face recognition were categorized as “normal”, “abnormal”, or “could not be evaluated”. Vision and hearing were “normal” (with or without glasses), “abnormal”, or “could not be evaluated”.

## 3. Results

Data were gathered on 651 subjects. The mean age was 13.7 years (range 5.2 to 27; 68% age < 16 years, 32% ≥ 16 years). The mean follow-up time from first contact with primary health care to the date of data extraction was 8.4 years (median 8.1, range 0.1 to 26). The demographic information is provided in Table 1. 

*Comorbid diagnoses.* Neuropsychiatric and psychiatric comorbid diagnoses were frequent. Out of 651 cases, 39% had ADHD/ADD, 23% anxiety symptoms, 16% conduct disorder, 14% autism spectrum disorder, 11% socioemotional problems, 8% mood symptoms, and 7% Tics/Tourette/obsessive-compulsive thoughts; 28% had two or more diagnoses. Moreover, 4.5% had severe reading comprehension disorder, 1.2% selective mutism, and 0.6% anorexia. Out of 206 adolescents (age over 16), 12% smoked regularly, 4.4% had an alcohol problem, 3.4% suffered from psychotic symptoms, 3% used illicit drugs, 2.4% had tried committing suicide, and 1.5% had delinquent behavior. 

*Evolution of diagnosis.* When studying the evolution of diagnosis between the first assessment and at discharge, the diagnosis of F83/F81.3 stayed the same in 50.5%. In 23%, the diagnosis evolved into ID (F70-F79), and in 2%, the diagnosis was removed as the patient performed the tests within normal limits. In 13.5%, the diagnosis of F83/F81.3 changed into a more specific learning disorder, such as specific language impairment (F80), developmental coordination disorder (F82), or ADHD (F90), and the opposite happened with 11% (change from a specific diagnosis to F83/F81.3). 

*Developmental and rehabilitation history.* The initial reason for contacting the primary care level was known for 552/651 (85%) cases. Language delay was the most prevalent symptom that worried parents (42%), followed by gross motor function delay (21%), learning difficulties (12.5%), and fine motor function delay (9%). The mean age when referred to a primary care level therapist was 3.4 years (median 3, range 0.1 to 15), and the mean age when referred to a neurodevelopmental clinic was 5.4 years (median 5, range 0.1 to 17). 

Out of 602/651 (92%) patients who reported on learning to walk, the mean age for attaining independent walking was 1.2 years (median 1.2, range 0.7 to 4), and in 64 (11%), walking was delayed over the age of 1.5 years. Out of 580/651 (89%) patients who reported on language development, the mean age for starting to speak sentences was 2.7 years (median 2.5, range 1.5 to 7), and in 101 (17%), speaking sentences was delayed over the age of 3 years. Nine children used Augmentative and Alternative Communication or sign language as their only communication. 

*Neurological development and neuropsychological profile.* The different neurological developmental domains (e.g., gross and fine motor function, coordination, play skills, social skills) were assessed by the multidisciplinary team´s therapists. The results from the physio- and occupational therapists´ assessments at the first referral and the abnormal findings (delay of 1.5 or more standard deviations below the mean of norm-referenced standardized tests) as frequencies and percentage for the individuals with available data are collected in Table 2. The most prevalent difficulties were noticed in eye-hand coordination skills, fine motor functions, and posture and balance.

**Figure 1:**
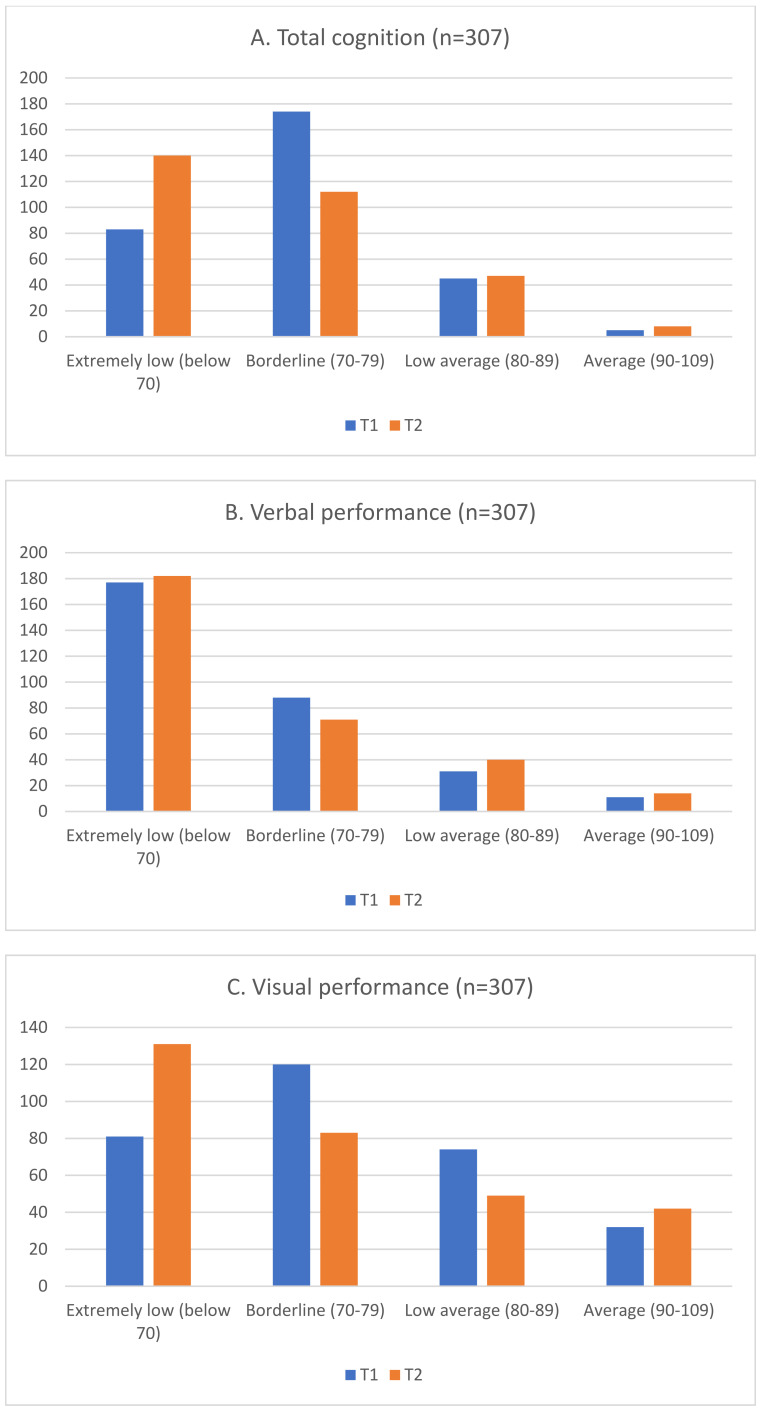

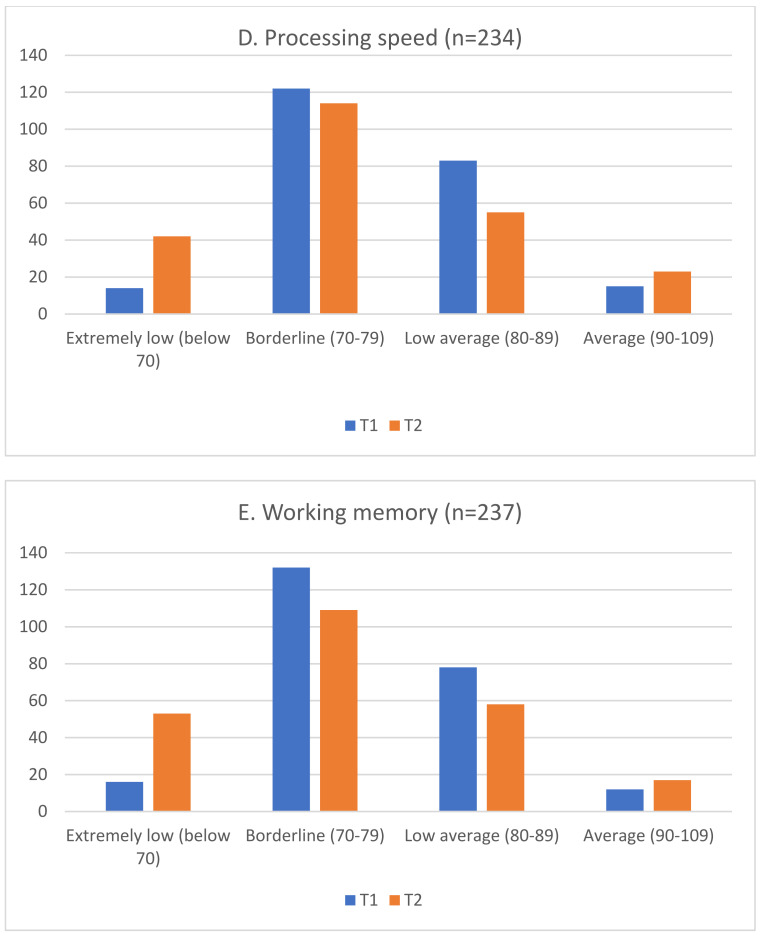
(**A**–**E**) Change in frequencies in total cognition, verbal and visual performance, processing speed, and working memory in the first (T1) and last (T2) neuropsychological assessments.

The total number of neuropsychological assessments per patient ranged from 1 to 7 (mean 2.9, median 3). The time-period between the first and last assessment in this study varied between 0.2 and 16 years (mean 3.8 years, median 2.4 years). At least two assessments were made for 352/651 (54%) participants. For the WPPSI-III/WISC-IV analysis, as to avoid learning effects, tests taken at least one year apart were chosen, comprising a total of 307/352 (87%) cases. Figure 1A–E show the frequencies in categories “Extremely Low”, “Borderline”, “Low Average”, and “Average” of the WPPSI-III/WISC-IV total cognition and subtests at assessment 1 (T1) and assessment 2 (T2)**.** The test results correlated negatively with the elapsed time between the two test sessions, which means that increasing the time between the two test sessions increased the probability of performing worse in the latter assessment. The Spearman´s ρ results for total cognition, verbal performance, visual performance, processing speed, and working memory were −0.31, −0.29, −0.27, −0.28, and −0.31, respectively. All correlations were statistically significant (*p* < 0.0001). 

Table 3 shows the results for the NEPSY-II neuropsychological subdomains of attention, executive functions, impulse control, reading skills, arithmetic skills, and abstract reasoning, and separately for conduct, self-esteem, and activities of daily living. At assessments 1 (T1) and 2 (T2) for those participants having data on both assessments. The proportion of participants having major difficulties increased from T1 to T2 in all subdomains, except for attention and impulse control. The increase was statistically significant in reading and arithmetic skills, abstract reasoning, and conduct. 

*Therapies received.* Different therapies were recommended for 634/651 (97%) children: occupational therapy for 70%, speech therapy for 66%, physiotherapy for 23%, music therapy for 7%, neuropsychologic rehabilitation for 6.5%, and horse riding for 2%. The therapies were reported to be fully accomplished in 63% and partly in 21.5% of cases, and for 1.5%, the therapy did not begin at all (for family or resource reasons). The number of concurrent therapies was 1 for 18%, 2 for 45%, and 3 for 18.5%. 

*Medication.* 294/651 (45%) participants used medication, and stimulants were most prevalent (32%), followed by antiepileptics (10%), risperidone (5%), other antipsychotics (4%), and antidepressants (2%); 4% used both a stimulant and an antipsychotic medication. Only seven children under the age of 7 received regular medication, among whom two were on stimulants. The reported stimulants (extended-release methylphenidate or lisdexamphetamine) were given once or twice daily, and the dosage ranged between 10 and 54 milligrams. However, because of irregular reporting on dosage, the exact mean dosages used as mg per kg could not be determined. Overall, 75% reported having benefitted and 81% were still continuing the stimulant medication, 31% had changed the stimulant to another type, and 19% had stopped stimulant use. 

*Referral to psychiatric or social services.* During the follow-up period, 29% were referred to a child psychiatrist, 10% to an adolescent psychiatrist, and 2% to an adult psychiatrist. In 7%, the need for psychiatric help continued from either childhood to adolescence or from childhood/adolescence into adulthood. In 8% social services, such as child welfare or home help services, were needed. Data on mental services use at the primary care level or at school were not available. 

*Need for support at school and special education*. The need for special pedagogical support (such as, remedial instruction, partial or fulltime small group teaching, adapting subjects and a modified school syllabus) and aids at school (such as, pictures or cards that help in communication, executive function, or transition situations; communication aids; instruction or aids that help with ADHD symptoms; small groups, or personal assistance) was frequent. At the time of the participants´ first psychological assessment, 468/651 (72%) needed educational support. Of those 128 participants attending elementary school, 60% studied using a normal syllabus, 38% used an individualized syllabus, 0.6% had their education organized by activity areas (special education for pupils with ID), and 11% needed a personal assistant. During the participants´ second psychological assessments, data on the school were available for 465/651 (71%) cases. Educational support was needed by 426/465 (92%) participants. 77% attended elementary school, of which 42% studied using a normal syllabus, 49% used an individualized syllabus, 8% studied with education organized by activity areas, and 3% needed personal assistance. 

*Progression into further education.* Data on progression into further education was gathered at the time of data extraction. At that point, out of 651 study participants, 5% attended preschool, 62% elementary school; 8% vocational school, 7% special vocational school, 0.8% university of applied sciences, and 0.8% attended college. Sixty-two (9.5%) completed elementary school, but data on their further education was not available due to such patients moving out of the area or not being reported in the patient charts. Four (0.6%) participants did not finish their elementary school, and 13 (2%) dropped out of their secondary level education studies (vocational, special vocational, or university of applied sciences). 

The neuropsychological profile was moved after Table 2 in the section with the neurodevelopmental results.

## 4. Discussion

BIF is a seldomly studied topic, and this population has remained as a marginal clinical entity with somehow vague diagnostic and therapeutic approaches [1,32]. Therefore, the aim of the present study was to explore the various neurodevelopmental characteristics and challenges in daily life of participants with BIF. 

There were several interesting findings. At the group level, in the WPPSI-III/WISC-IV total cognition, verbal and visual performance, processing speed, and working memory the results in each subtest declined with time between the two assessment sessions T1 and T2. This was noticed as a change in the frequencies of participants in “Extremely low” at T2 exceeding the frequency of “Borderline” at T1 in total cognition and visual performance. This suggests that the visual performance difficulties become more apparent as the child grows and demands in testing the different domains increases but the child is not able to catch up. Also, at the first assessment, most of the participants already performed at the level of “Extremely low” in the verbal performance, while the visual performance level remained “Low average” or “Borderline”. However, by the last test, the visual performance subtest had dropped to an “Extremely low” level, as well. This same pattern is seen in the processing speed and working memory. The working memory tests gradually become more difficult with age groups, starting from repeating simple sentences and extending to remembering abstract concepts. Processing speed requires fine motor precision and motor and executive planning, which all are demanding for individuals with BIF [31,33].

Likewise, there was a tendency of decline over the long run in executive functions, reading and arithmetic skills, abstract reasoning, conduct, self-esteem, and activities of daily living. These increasing difficulties indicate that individuals with BIF have marked deficits in the processing and integration of verbal and visual memory information, executive functions and working memory, and more complex reading and arithmetic comprehension. Additionally, these difficulties become more evident as the expectations and demands of academic achievements and task complexity grow. These findings parallel the clinical notions in patients with BIF [31,33].

Delays in language and gross motor functions were the most prominent initial reasons for parents to contact primary health care. Parental concern was found to be a useful approach to screening for the early detection of developmental delays, especially in language and motor development, whereas cognitive and behavioral problems were less likely to be detected by the parents [34]. In their systematic literature review, Peltopuro et al. [1] found an early delay in mental processing, talking, or motor development to be connected to BIF. Due to the lack of a comparison group of normally developing children and adolescents, we were not able to run any statistical analyses to determine factors for predicting the diagnosis of BIF. However, some findings may point towards more serious global delays, such as early emerging difficulties in language and motor development, executive functions, or learning the activities of daily living prior to school age and delays in academic skills (reading and arithmetic skills) and abstract reasoning at school age. 

At the time of data extraction, only in 15.5% the cognitive performance had improved so that the diagnosis was either removed or changed into a specific diagnosis. In 62%, the diagnosis of F83/F81.3 either stayed the same, or a more specific diagnosis was changed into F83/F81.3. Notably, in 23%, the diagnosis evolved into ID (F70-F72). Literature on the prevalence of BIF diagnoses changing into ID is rare. In their follow-up study of 45 children diagnosed with global developmental delays at age 2, Dornelas et al. [35] found that 4.4% of the participants were diagnosed with ID at school age, while 33% developed cognitively normally. Peltopuro et al. [29] estimated that at least 11% of their study participants with BIF were diagnosed with ID later in life. Our results not only exceeded these figures, but we suspect that the quantity of ID diagnoses might be even greater if the follow-up period were longer. Therefore, it is important to repeat the neuropsychological testing of cognitive and adaptive functions before the age of 18 to capture those who meet the ID criteria and offer these individuals the legitimate services they need. 

In the present study, comorbid neuropsychiatric and psychiatric diagnoses were frequent. A related finding was that a total of 41% of the study participants were referred to psychiatric services. Most of the studies assessing the prevalence of comorbidities and the use of psychiatric referrals were done either with children and young people with ID or with combined groups of children suffering from BIF and ID [7,17,21,24,25,28,29]. In children with borderline to moderate ID, Dekker et al. [24] found 22% to have anxiety, 4.4% to have a mood disorder, and 25% to have a conduct disorder. About 27% of the diagnosed children had received mental health care during the year before the study interview. Handen et al. [17] and Peltopuro et al. [29], respectively, found 22% and 19% of their study participants with BIF to need psychiatric inpatient care. Our results in both psychiatric referrals and comorbidity frequencies parallel these aforementioned studies. The literature indicates a risk ratio of 2.4 for having a psychiatric diagnosis [28] and 3 to 3.4 for psychiatric inpatient treatment [20,29] compared with the typically developing general population. Conversely, it is important for psychiatric clinicians to recognize the possibility of BIF or ID among those being admitted to psychiatric clinics. In their adult inpatient cohort, Nieuwenhuis et al. [36] found almost 44% of the sample to have BIF or mild ID.

It has been shown in the literature that in 65–75% of children with ID, emotional problems tend to persist or even increase over time, while the rate of behavioral problems declines [20,21]. This phenomenon may also apply to children with BIF. In our study, the appearance of conduct problems remained stable over time. Unfortunately, we had no data on parental distress and family dysfunction, or how early the emotional symptoms started, which are all factors considered to predict the development of psychopathology [20,21]. Due to the study design, the figures on alcohol or drug use and the use of social services, such as child welfare or home help service, are likely to be underreported, but they highlight the need to recognize the socioemotional and mental burden of cognitive difficulties and the importance of cooperation between pediatric neurologists, child and adolescent psychiatrists, child psychologists, and social services. 

Stimulants are the treatment of choice in normally developing children and adolescents with ADHD, and a good clinical response rate of 65 to 75% in the reduction of ADHD core symptoms has been reported [37,38,39]. Individuals with BIF and ID respond to methylphenidate, though the response rate may be more modest, varying from 40 to 70% [17,38,40,41]. Additionally, vulnerability to side effects with the doses normally used in ADHD may be subtle and heterogeneous [38,40,41]. In the Handen study [17], 70% of children aged 7 to 14 years old with BIF and ID continued their stimulant medication for 1 to 5 years, indicating both a need for and a sufficient response to the medication. In our study, 75% felt that stimulants were helpful, and 81% continued the medication at the time of data extraction. Information on side effects was not gathered, but 31% had to change the first stimulant to another label. Bearing in mind the limits of gathering data using a retrospective study design, our results suggest that stimulants work well and that the adherence to treatment is good in ADHD for children and adolescents with BIF.

There was a two-year gap between the mean ages for primary health care and secondary level neurodevelopmental clinic referrals. However, in our national health care system, the aim is for primary care physicians and community health nurses to send children to therapists for evaluation or families to counseling as soon as the problems are noted. Thus, this gap period does not necessarily indicate a lack of support. A total of 97% of the study participants had received some sort of therapy, mostly two concomitant therapies, reflecting both the need for and the amount of developmental support. We were not able to obtain data on how long the therapies lasted, but generally, the focus shifted from speech, physio, or occupational therapy in childhood to neuropsychologic rehabilitation and educational support in school at school age. 

Educational support in kindergarten or grade school was needed by 72 to 92% of the study participants. This support could be, e.g., pictures or cards that help in communication, executive function, and transition situations; communication aids; instruction or aids that help with ADHD symptoms; small groups, or personal assistance. The need for special pedagogical support (remedial instruction, partial or fulltime small group teaching, adapting subjects (e.g., in math, reading, or languages) and a modified school syllabus increased with age. Generally, in Finland, the need for special pedagogical support was 23% among elementary school pupils and 11% among second-grade students in 2021 [42].

Notably, at the time point of data extraction, only 0.6% of the participants for whom data existed had discontinued their time at elementary school. The majority of those graduating elementary school continued their education at ordinary or special vocational schools, but the risk of dropping out during secondary-level studies (2%) appeared to increase. This result may indicate that the support given at elementary school level is sufficient to enable pupils to attain their academic goal of graduating. At the college/vocational school stage, support may decrease and be unable to meet the needs of students as their challenges grow and educational surroundings change. According to Finnish national statistics, in 2020, 6.8% of all secondary students discontinued their studies, while 13.3% in vocational school, 7.2% in universities of applied sciences, and 3.6% in college discontinued their studies [43]. Concomitantly, this age is important for detaching oneself from parents and home, gaining independence to take care of one’s own matters, building new relationships, and orientating oneself towards a future occupation, thus demanding quite a variety of skills. Data beyond elementary school were limited to those who continued at neurological or student medical clinics, but there were indications highlighting an increased risk of unemployment, as well as difficulties attaining and keeping a job and receiving disability pensions. The literature shows that the disability pension rates are 2.7 times greater for individuals with BIF and 6.9 times greater for individuals with ID compared to the general population; moreover, many such individuals work in unskilled or semiskilled jobs with a lower income [1,29].

The limitations of this study are that due to the nature of its retrospective design, we did not have a control group. The data were collected from a single neuropediatric setting. Therefore, generalization to other populations may be limited. Collecting and analyzing data retrospectively also poses difficulties due to incomplete documentation, varying follow-up length, and missing or unavailable data, such as data on custody, fostering, the need for housekeeping aid or other social support, and employment. In addition, we could not gather information on the probable predictive or preventive factors that could have been useful for planning interventions. However, we feel that our study population reflects common practice.

## 5. Conclusions

Some early emerging developmental signs could help diagnose and provide support for BIF, such as difficulties in language and motor development, executive functions, or learning the activities of daily living prior to school age, as well as delays in academic skills (reading and arithmetic skills) and abstract reasoning at school age. Notably, in 23% of BIF patients, the diagnosis evolved into ID. It is important to follow up and support individuals with BIF as their risk for being left behind in the society is increased. Also, it would be important to repeat the neuropsychological testing of cognitive and adaptive functions before graduating elementary school as to capture those who meet the ID criteria.

## Figures and Tables

**Table 1:** Patient demographics of the 651 study participants.

Parameter	Patients *n* (%)
Male	411 (63)
Female	240 (37)
Living in:	
Nuclear family	322 (49)
Single parent family	215 (33)
Foster family	57 (9)
Blended family	55 (8)
Young adults still living with parents	6 (0.9)
Shelter home	1 (0.2)
Dating or married	16 (2.5)
Mother language:	
Finnish/Finnish–Swedish	551 (85)
Other (21 different languages)	100 (15)
Vision deficiency *	39 (6)
Hearing impairment	20 (3)
Information on head circumference (*n* = 455)	
Microcephaly	42 (9)
Macrocephaly	16 (4)
Information on weight (*n* = 489)	
Overweight (>+20% of normal)	129 (26)
Underweight (<−20% of normal)	15 (3)

* Two totally blind; squinting, astigmatism, or mild myopia cases not included.

**Table 2:** Abnormal findings in different neurological developmental domains for those individuals with data available.

Developmental/Social Skills	Abnormal, *n* (%)	Data Available, *n* (%)
Gross motor function	248 (40.3)	615 (94.5)
Fine motor function	445 (72.7)	612 (94)
Posture and balance	399 (65.5)	609 (93.5)
Eye–hand coordination skills	476 (77.9)	611 (93.9)
Sensory integration skills	235 (41.7)	564 (87)
Playing alone	55 (9.5)	582 (89)
Playing side by side	69 (11.9)	582 (89)
Playing interactively	215 (36.6)	587 (90.2)
Having friends	198 (33.1)	598 (91.9)
Catching social clues	270 (45.9)	588 (90.3)
Recognizing facial expressions	64 (14.2)	450 (69.1)
Social participation	249 (41)	607 (93.2)

**Table 3:** The proportion of participants having major difficulties (frequency and percentage) in the assessed NEPSY-II neuropsychological subdomains, and conduct, self-esteem, and activities of daily living compared between assessment 1 (T1) and 2 (T2). The number of patients with data on both assessments within the given subdomain is shown in “*n*”-column.

Subdomain Evaluated	*n* *	T1 *n* (%)	T2 *n* (%)	*X* ^2^	*p*
Attention	342	165 (48.2)	140 (40.9)	3.408	0.0649
Executive functions	335	166 (49.6)	186 (55.5)	2.161	0.1416
Impulse control	328	98 (29.9)	85 (25.9)	1.091	0.2962
Reading skills	122	49 (40.2)	89 (73)	25.371	0.0000
Arithmetic skills	109	26 (23.9)	51 (46.8)	11.566	0.0007
Abstract reasoning	202	59 (29.2)	100 (49.5)	16.594	0.0000
Conduct	337	177 (52.5)	208 (61.7)	5.452	0.0195
Activities of daily living	321	37 (11.5)	43 (13.4)	0.357	0.5502
Self-esteem	169	22 (13)	30 (17.8)	1.114	0.2913

* Frequency of patients with corresponding information for both T1 and T2.
